# Child marriage among Somali refugees in Ethiopia: a cross sectional survey of adolescent girls and adult women

**DOI:** 10.1186/s12889-021-11080-5

**Published:** 2021-06-02

**Authors:** Shatha Elnakib, Kara Hunersen, Janna Metzler, Hailu Bekele, W. Courtland Robinson

**Affiliations:** 1 Johns Hopkins Bloomberg School of Public Health, Baltimore, USA; 2 Women’s Refugee Commission, New York, USA; 3 International Medical Corps, Addis Ababa, Ethiopia

**Keywords:** Displacement, Humanitarian context, Child marriage, Adolescent health, Conflict, Sexual and reproductive health, Ethiopia, Somali refugees

## Abstract

**Background:**

Despite child marriage receiving increased attention over the past two decades, research on child marriage in humanitarian settings remains scarce. This study sought to quantify child marriage among Somali adolescent girls residing in Kobe refugee camp in Ethiopia and to identify its correlates and consequences.

**Methods:**

A cross-sectional survey was conducted using multi-stage cluster-based sampling with probability proportional to size. We randomly sampled households that have at least one female aged 15–49 and at least one adolescent female aged 10–19. In addition to calculating the proportion of girls married under age 18, we used survival methods – namely Kaplan Meier graphs and Cox proportional hazard models – to identify risk factors associated with child marriage in this context. We also used descriptive statistics to describe marital age preferences among female adults and presented measures of important sexual and reproductive health indicators among married adolescent girls.

**Results:**

A total of 603 adult women were surveyed and a household roster was created with information on 3319 household members, of whom 522 were adolescent girls aged 15–19. Of those, 14% were currently married (95% Confidence Interval [CI] 0.11–0.18), and 11% were ever married under age 18 (95% CI 8–15%). Several variables were found to be significantly associated with hazard of child marriage including schooling, sex and employment status of head of household, as well as number of girls under age 18 in the childhood home.. Adult women tended to incorrectly identify minimum legal age at marriage and preferred low marital age for boys and girls – particularly in households of child brides. Among married adolescent girls, contraceptive use was very low (11%; 95% CI 4.94–22.40), and early childbearing was common (60%; 95% CI 45.56–72.89).

**Conclusions:**

This research contributes to the evidence base on child marriage in humanitarian settings. Insights generated from this study have the potential to inform programs and interventions aiming to prevent and mitigate the impacts of this harmful practice.

**Supplementary Information:**

The online version contains supplementary material available at 10.1186/s12889-021-11080-5.

## Background

Child marriage, defined as marriage or informal union under the age of 18, is a human rights violation disproportionately affecting girls in developing countries [1]. Although child marriage has decreased over the past twenty years, it remains ubiquitous in many parts of the world, and around 39,000 girls become child brides every day [2]. In Somalia, the practice is pervasive and according to DHS 2020, 36% of women aged 20–24 were married under age 18 and 18% of 15–19 year old girls are currently married [3].

Child marriage has serious public health and social consequences for young women and their children. Where child marriage is common, adolescent birth rates tend to be highest and complications arising from early childbearing represent a major cause of mortality [4–6]. Several studies have demonstrated the association between child marriage and adverse mental health outcomes such as depression [7], stress [8, 9], and suicidality [10]. Married adolescents are less likely to use family planning [11, 12], have reduced access to healthcare services including antenatal and postnatal care [11, 13], and tend to have poorer self-reported health compared to their unmarried counterparts [11, 14]. Research suggests that due to spousal age gaps that characterize child marriages, girls’ autonomy and bargaining power in the marital home are diminished, and risk of intimate partner violence is elevated [15–18] . Child marriage has also been shown to have intergenerational effects, with important consequences for child development and health [19].

In contexts of conflict and protracted displacement, women and girls are at heightened risk of sexual violence [20]. The confluence of poverty, violence, and the breakdown of services and community structures due to displacement may place girls at increased risk of child marriage. In Bangladesh, Somaliland and Niger, a World Vision study identified child marriage as a common practice in response to conflict and natural disaster [21]. A report by Human Rights Watch highlighted the impact of political turmoil and conflict on the practice of child marriage in Yemen [22]. Research among internally displaced persons and Congolese refugees in Uganda also indicates that structural changes related to displacement coupled with shifts in the economy and family relations contributed to the practice of early marriage [23]. Yet, empirical evidence supporting the claim that child marriage increases in contexts of displacement is scant and the body of literature documenting the drivers of child marriage in humanitarian settings is limited [23–25]. Moreover, although some reports indicate that child marriage may increase in humanitarian settings and in response to conflict, this is not universally the case. In some contexts, conflict has led to a postponement of marriage among adolescents [26–28], implying that conflict may engender different responses and coping strategies.

**Fig. 1:**
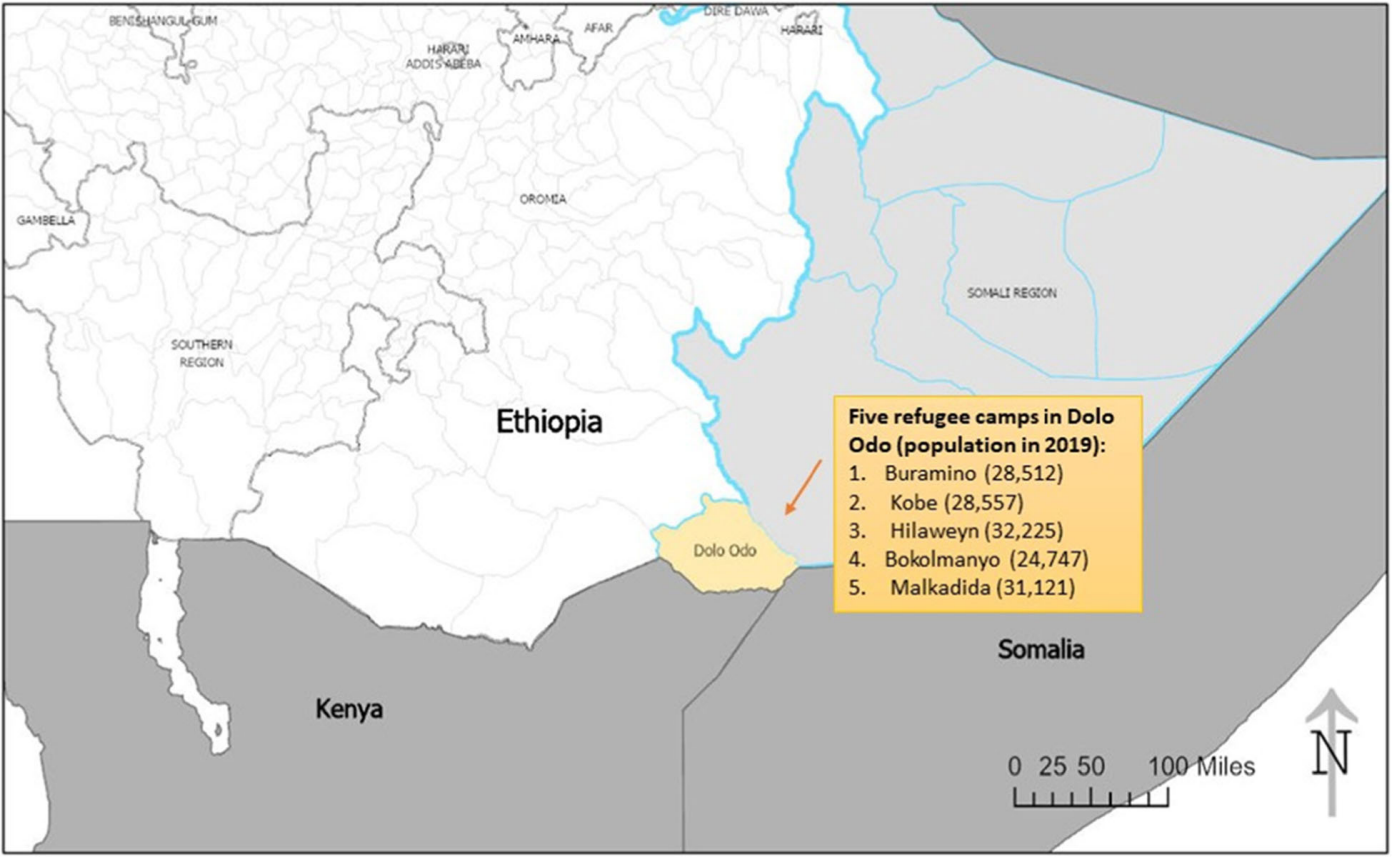
Map of Ethiopia with Dolo Odo refugee camps’ population size, generated using ArcGIS Pro

Little is known about the scale of child marriage among Somali refugees displaced to Ethiopia’s southeastern Dolo Odo corridor, where minimum age at marriage is 18 but not always consistently enforced. Predominantly rural and pastoral, Somali refugees residing in Kobe camp have fled internal conflicts that have spanned over two decades [29]. In July 2011, in addition to ongoing conflict, the UN declared the worst famine in a generation in south-central Somalia after a severe drought beset the country leading to the “Horn of Africa Crisis.” [30] The drought and ongoing conflict have had a devastating impact, forcing 1.5 million persons to flee to safer areas and camps in neighboring countries like Ethiopia and Kenya. In Ethiopia, the Dolo Odo refugee complex hosts Somali refugees and is considered one of the largest refugee complexes in the world [31]. In 2019, the population in Dolo Odo refugee complex – which comprises 5 camps, Buramino, Kobe, Hilaweyn, Bokolmanyo and Malkadida camps – is estimated to have reached 142,000 persons (Fig. 1) [32]. The Kobe refugee camp alone hosts a total of 28,557 refugees, half of which are female and around 68% are under 17 years [33].

Given that child marriage is widespread in Somalia and in light of pressures presented by displacement, there is a pressing need for data on the marriage practices and customs of Somali refugees living in this context. This study presents findings from a household survey conducted among Somali refugees residing in Kobe refugee camp. The aims of the study are two-fold: 1) to quantify the proportion of girls married under age 18 among Somali refugees in the Kobe camp; and 2) to investigate drivers and consequences of the practice for adolescent girls in this setting.

## Methods

### Overview of design

**Fig. 2:**
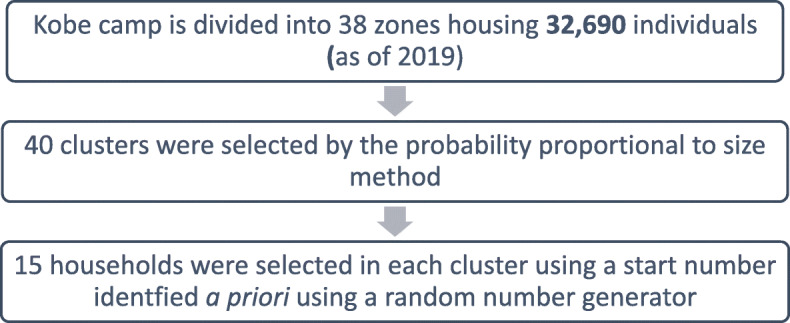
Sampling design

The study employed a multistage cluster sample survey design (Fig. 2), which is a cost- and time-effective probability sampling method. The study population comprised an estimated population of 5706 households within 38 zones in Kobe camp. A total of 40 clusters – representing blocks nested within zones and consisting of 15 households each – were selected using the probability proportional to size method to create a self-weighted sample. We sampled households within each cluster that have at least one female adult and at least one adolescent female 10–19. For a sampling frame, estimates of the number of households per zone were based on UNHCR population data. Absent data on child marriage prevalence, a prevalence of 50% was presumed to maximize sample size. The margin of error was set at ±5% and the design effect for cluster sampling was assumed to be 2. Accordingly, a sample size of approximately 800 households with at least one woman between the ages of 15 and 49 who has lived at least 1 m in the household was needed. Due to budget constraints, however, only 600 households were sampled. We used a random start number and the appropriate sampling interval until 15 eligible households were reached in each cluster (given the selection of 40 clusters). Within each selected household, one interview was conducted with either the female head of household or a female adult who has been living in the household for at least 1 m in the last year. This individual was asked to provide a roster of all household members, including all married and unmarried females between the ages of 10 and 19 (including any who moved out of the household in the last year). From the roster of married and unmarried females between the ages of 10 and 19, the interviewer randomly selected one married female (if more than one) and/or one unmarried female (if more than one) to interview more in depth about her experiences of, and/or attitudes about, child marriage.

The criteria for inclusion into the survey were the availability of at least one female aged 15–49 who has been residing in the household for at least 1 m in the previous year. If not present, any woman in the household who is 19 and over or married between ages 15–17 was interviewed instead. The household had to also include at least one adolescent female aged 10–19 currently living in the household. Ability and willingness to participate in the interview and provision of informed verbal consent as well as parental approval and assent for unmarried girls under age 18 were additional inclusion criteria.

### Data collection

The study team comprised 16 enumerators, a research coordinator representing a partnership between Johns Hopkins University and Women’s Refugee Commission, two IMC staff members, two program managers from the Kobe Women’s Center, two field supervisors, and two interpreters (who supported the research coordinator and IMC staff in communications with the enumerators). Enumerators were Somali females aged 18–25 recruited from the local refugee population. They were all fluent Somali speakers and knowledgeable of the camp community. All enumerators received a 5-day training which was led by the research coordinator who was on site for the entire study period. Training topics included study background, research ethics, working with vulnerable populations, and communication skills. Two days were dedicated to explanation of the research protocol, as well as in-depth review of the quantitative tools. The final two days focused on tablet use and role play, along with a pilot of the survey in Kobe refugee camp. The research coordinator was present with the study team in the refugee camp each day of data collection, with morning meetings to assign clusters and set daily goals, and afternoon meetings to address any challenges and provide support. During the first week, oversight included periodic visitation by the research coordinator of data collectors in the field to address any issues, as well as daily quality checks of collected data. In the following weeks, supervisors remained with data collectors in the field and the research coordinator checked tablets at the end of each day, uploaded the data, and monitored it for any inconsistencies or missingness.

Using a recruitment script, the enumerators introduced the study to a potentially eligible household and obtained information from a household representative about household composition (sufficient only to determine household eligibility). If the household was deemed eligible and the household representative expressed willingness to be possibly consented, the study team member either identified a time to return and consented the household into the study or proceeded directly to the consent process.

### Survey questionnaire

Two survey questionnaires were developed incorporating validated questions from the Demographic Health Survey and MICS and adapted to this setting through collaboration with the IMC study team based in Dolo Odo (Supplementary files 1 and 2). The questionnaires were piloted in the refugee camps to ensure cultural sensitivity and applicability.

For adult females interviewed in the selected household, the survey questionnaire collected information on the roster of household members, their age, gender, age at marriage and a number of other characteristics. Additionally, demographic data were collected on the household including age and sex of household members; time in current residence; previous residence and reasons for moving; place of origin; education; occupation of household head as well as data concerning perceptions of marriage..

After the household survey, the interviewer proceeded to interview one or up to two females residing in the same household. Questions in this questionnaire included number of years of education; out-of-household work experience (if any) in the last year; perceptions of marriage; risk factors for child marriage; marital status; and exposure to interventions aimed at preventing child marriage. The results presented in this study use both the household roster and adolescent interview data.

### Data analysis

Data analysis was performed using Stata 15 (StataCorp, College Station, TX), using “svyset” to specify the survey design characteristics and calculate Taylor linearized variance estimates. Because the rosters elicited from the heads of household included a greater number of girls in the age range 15–19, we relied on roster data for most of the analysis, unless otherwise noted. Proportion of child marriage was estimated and 95% confidence intervals (CIs) were calculated. Two measures were calculated: the proportion of girls 15 to 19 years of age currently married or in union, and the proportion of women aged 20–24 first married or in union before age 18. The first indicator provides a more current view of child marriage but does not account for right censoring and thus may underestimate the true extent of child marriage because girls may not have completed time at risk. The second indicator avoids right censoring because all women completed time at risk, but the data offers a retrospective or lagged view of marriage practices. We also presented the proportion of women aged 18–19 who were married before age 18 to address issues inherent in both indicators.

To account for right censoring, subsequent analysis used survival methods. Kaplan-Meier estimates of marriage-free probabilities were calculated for each independent variable and were compared using the log rank test. Cox Proportional hazards models were used to estimate hazard ratios of child marriage associated with selected independent variables and their confidence intervals. Survival time was defined as the age of respondent at first marriage, while for those who were unmarried at the time of the survey, survival time was defined as their age at the time of the survey. A censoring variable was created such that girls who were either not married at the time of the survey, or who reached age 18 without getting married were censored. The proportional hazards assumption was assessed using Schoenfeld residuals. Finally, descriptive analysis of a set of indicators pertaining to sexual and reproductive health knowledge and outcomes was done using survey data with child brides interviewed in the study.

### Independent variables

We focused on variables related to socio-demographics, family characteristics, and displacement variables and used these variable groups separately in the Cox regression models due to sample size considerations. **Sociodemographic variables** included reported current *age* – modelled as a continuous variable, *education status* (ever attended school vs never attended school), *ethnicity* (Darod, Digil and Mirifle, Dilir, and other) as well as *type of residence* in Somalia (urban vs rural). **Family characteristics** included the *sex* of the head of household,^1^*number of girls under age 18* residing in the original household (none, one, two or more) – to account for potential sister effects [34] – as well as *whether the mother was married as a child.* **Displacement-related variables** included *number of times a household moved* in the past 12 months (none, once, two or more),*duration in years in the camp* – modelled as a continuous variable*, and unemployment of head of household (currently working* vs *not currently working)*.

## Results

### Prevalence of child marriage

A total of 603 adult women were surveyed and a household roster was created with information on 3319 household members, of whom 882 were females in the age group 10—19. In addition, 650 adolescent girls were interviewed from the same households. Of the female adults interviewed in this study, 66.44% reported getting married under age 18 (95% Confidence Interval [CI] 61.74–70.84%). In contrast, among 15–19-year-old adolescent girls in the roster, 14.23% were currently married (95% CI 10.87–18.41%), and 11.15% were married under age 18 (95% CI 8.23–14.95%). Among those aged 18 and 19 who have completed time at risk for child marriage, 25.29% were married under age 18 (95% CI 18.8–33.1%). Women ages 20–24 in the household roster were too few (*n* = 13), and 8 of them were married under age 18 (Percent = 46.15% 95 CI 19.90–74.73%).

### Correlates of child marriage

Table 1 shows the characteristics of adolescent girls included in the roster stratified by child marriage status. The first column presents data for girls married under age 18 and the second column presents data for girls who were either unmarried or married above age 18 at the time of the survey. The median age is 18 among girls married as children and 17 among girls never married as children. A greater proportion of girls who were not married under 18 attended school (90.26% compared to 60.34% among ever married girls) and attained higher education levels. A higher proportion of both groups came from rural as opposed to urban areas in Somalia. Both groups experienced protracted displacement, with respondents residing in Kobe camp for a median of 9 years. In terms of family characteristics most girls married as children came from households in which female respondents stated they were the heads of households (77.59% vs 44.59% among girls not married as children). Households of girls who were married under age 18 had an average of 1 female member under age 18; compared to 1.5 in the other group. Across both groups, the vast majority belonged to households where the head of household (HH) was reportedly not currently working. Less than 10% reported disability in the household across both groups, and the majority reported not moving in the past 12 months. The median age of spouses of girls married under age 18 was 25 years old.

**Table 1:** Characteristics of adolescent girls (aged 15–19) in the roster *(N = 522)*

	Ever married before age 18		Never married before age 18	
**Variable**	(*N* = 58; 52 households)		(*N* = 462; 415 households)	
*I. Sociodemographic variables*				
**Age of respondent, median, IQR**	18	1	17	3
**Ever attended school,** ***N*, %**				
Yes	35	60.34	417	90.26
No	23	39.66	45	9.74
**Highest level of school,** ***N*, %**				
None	0		5	1.20
Primary	32	91.43	322	77.22
Secondary	3	8.57	88	21.10
Higher	0		2	0.48
**Ethnicity,** ***N*, %**				
Darod	16	27.59	207	44.81
Digil and Mirifle	22	37.93	143	30.95
Dilir	9	15.52	41	8.87
Other	11	18.97	71	15.37
**Residence in Somalia,** ***N*, %**				
Urban	25	43.1	186	40.26
Rural	33	56.9	276	59.74
**Age of spouse, median, IQR**	25	8		
*II. Family variables*				
**Number of households that are female-headed,** ***N*, %**	45	77.59	206	44.59
**Education of adult female,** ***N*, %**				
None	52	89.66	442	95.67
Preschool	0	0	4	0.87
Primary	5	8.62	13	2.81
Secondary	1	1.72	2	0.43
higher	0	0	1	0.22
**Number of girls in the household below age 18, mean, sd**	1.02	0.96	1.52	1.09
*III. Displacement and vulnerability*				
**Disability/ Injury,** ***N*, %**				
Yes	4	6.90	25	5.41
No	54	93.10	437	94.59
**Number of times a household moved in the past 12 months,** ***N*, %**				
0	41	77.36	356	80
1	9	16.98	63	14.16
2+	3	5.66	26	5.84
**Duration in camp, median, IQR**	9	1	9	1
**Marriage occurred after displacement,** ***N*, %**	55	95	12	100
**Head of household currently working,** ***N*, %**				
Yes	49	84.48	342	74.03
No	9	15.52	120	25.97

As shown in Table 2, several variables were significantly associated with hazard of marriage under age 18 among girls aged 15–19. Simple Cox regression revealed that hazard of marriage increased for older girls (HR 2.0, 95% CI 1.6–2.5) and for girls who came from female-headed households. Indeed, girls were more than 4 times at increased hazard of marriage if they came from households headed by females vs males (HR 4.5, 95% CI 2.4–8.4). Girls from Digil and Mirifle sub-clans had higher hazard of child marriage compared to those from Darod clans (HR 1.8, 95% CI 1.0–3.2) and girls belonging to households where the head was unemployed were two times more likely to enter into marriage under age 18 (HR 2.1, 95% CI 1.1–3.9).

**Table 2:** Crude and adjusted Cox regression of explanatory variables for marriage under age 18 among girls aged 15–19

	Simple Cox regression	Model 1: Sociodemographic characteristics	Model 2: Family characteristics	Model 3: Displacement-related variables
	HR (95% CI)	aHR (95% CI)	aHR (95% CI)	aHR (95% CI)
***I. Sociodemographic***				
Age	2.00 (1.60–2.51)***	1.77 (1.44–2.18)***	–	–
Ever in school (ref = no)	0.25 (0.14–0.45)***	0.34 (0.17–0.67)**	–	–
Ethnicity (ref = Darod)				
Digil and mirifle	1.82 (1.02–3.24)*	1.38 (0.68–2.80)	–	–
Dilir	2.22 (0.89–5.52)	1.97 (0.86–4.48)	–	–
Other	2.10 (0.73–6.04)	2.09 (0.84–5.22)	–	–
Type of residence in Somalia (ref = urban)	0.93 (0.56–1.55)	1.30 (0.70–2.41)	–	–
***II. Family***				
Sex of head of household (ref = male)	4.49 (2.39–8.43)***	–	4.83 (2.64–8.82)***	–
Number of girls under age 18	0.62 (0.40–0.99)*	–	0.58 (0.39–0.86)**	–
Female adult married as a child (ref = No)	1.16 (0.65–2.05)	–	1.83 (0.97–3.45)	–
***III. Displacement***				
Number of times household moved (ref =0)				
1	1.19 (0.68–2.07)	–	–	1.07 (0.61–1.89)
2 or more	1.02 (.21–5.03)	–	–	1.09 (0.22–5.38)
Duration in displacement (in years)	0.93 (0.85–1.02)	–	–	0.92 (0.84–1.02)
Unemployment of head of household (Ref = currently working)	2.08 (1.11–3.90)*	–	–	2.19 (1.04–4.64)*

*Significant at *p* ≤ 0.05; **p ≤ 0.01; ***p ≤ 0.001;

Moreover, girls who come from households with multiple girls under age 18 had a lower hazard of entry into child marriage (HR, 0.6 95% CI 0.4–1.0). School attendance was inversely associated with hazard of child marriage, with those who ever attended school at 75% reduced hazard of marriage (HR 0.25, 95% CI 0.1–0.5).

After adjusting for other variables in model 1, the association between age and hazard of child marriage remained significant – although attenuated – and so did the association with school attendance. Ethnicity was no longer associated with hazard of marriage and neither was type of residence.

In model 2 which explores family characteristics, girls from female-headed households had higher hazard of child marriage. Additionally, girls from households with higher number of girls under age 18 had a lower hazard of child marriage.

In terms of displacement-related characteristics, neither number of times that a household moved in the past 12 months nor the duration in displacement were found to impact hazard of marriage, after adjusting for other covariates. However, unemployment of head of household continued to be associated with child marriage hazard.

### Marital age and decision-making: adults’ perspectives

Adult females were asked about their perception of the ideal timing of marriage for girls and boys (Table 3). Women residing in households where a girl married under age 18 were more likely to report a lower marital age preference compared to women from households without a child bride (*p* = 0.036). On average, respondents tended to report higher marital age preference for boys; however, those residing in households of child brides preferred a younger age at marriage for boys compared to those without child brides (*p* = 0.042). Although a similar pattern can be observed regarding legal age at marriage, the difference between households with child brides and those without child brides was not statistically significant.

**Table 3:** Views on age at marriage among households with girls ever married as children and never married girls

Indicator	Mean score +/− sd		*P* value
	Ever married girls’ households	Never married girls’ households	
Perception of ideal age of marriage for girls	16.5 +/− 2.14	17.07 +/− 2.45	0.036
Perception of ideal age of marriage for boys	18.41 +/− 2.59	19.19 +/− 2.99	0.042
Perception of legal age at marriage for girls	16.65 +/− 2.56	17.21 +/− 1.88	0.21
Perception of legal age at marriage for boys	20.47 +/− 2.85	20.89 +/− 2.65	0.28

Around 73% of women stated that boys are the main decision makers when it comes to age at marriage. In contrast, only 64% considered girls as decision makers as well (*p* < 0.05) [data not shown]. In terms of what influences decisions about the age at which people get married, 98% of respondents cited religion as the main driver, and the remaining respondents cited reasons related to family honor and tradition. Most participants (95%) also stated that marriages were most often formalized by religious leaders not government officials.

### Child brides’ SRH knowledge practices and outcomes

In Table 4, we present descriptive measures for indicators of sexual and reproductive health knowledge and outcomes among child brides surveyed in this study. A total of 487 girls aged 15–19 were interviewed, of whom 13.76% were currently married (95% CI 10.54–17.76%) and 11.09% were married under age 18 (95% CI 8.36–14.56%). Most married adolescent girls reported knowledge of how to get pregnant and how to keep healthy during pregnancy, but knowledge of dangers of early childbearing and contraception was less common. This is further reflected in the finding that most girls reported initiation of childbearing, with the average number of children for girls in this age group being 1.21 and average age of birth at 16.85. Contraceptive use was very low in this sample. Only 11% of girls reported contraceptive use – mostly oral contraception (83.33%) and condoms (16.67%), despite that most girls reported ever using health services provided in Kobe camp (85.4%).

**Table 4:** Indicators for SRH knowledge, practices and outcomes among girls aged 15–19 married before age 18

Indicator	Married under age 18 (*N* = 54)	
	*n*	% (95%CI)
**Knowledge of SRH**		
Knowledge on how to become pregnant	39	95.12% (81.45–98.86)
Knowledge on how to keep healthy during pregnancy	38	92.68% (72.23–98.40)
Knowledge of dangers of early childbearing	26	63.41% (44.63–78.85)
Knowledge on contraception	25	60.98% (39.26–79.07)
**Practices and outcomes**		
Ever use of a health facility	35	85.37% (71.78–93.04)
Use of contraceptive method	6	10.91% (4.94–22.40)
Ever given birth	33	60.00% (45.56–72.89)
Death of a child	1	3.13% (0.37–22.12)
Number of children (mean +/− sd)	1.21	+/−0.60
Age at first birth (mean +/− sd)	16.85	+/− 2.95

## Discussion

This study sheds lights on marriage practices of younger cohorts of Somali refugees displaced to Kobe camp in Ethiopia. While some studies on child marriage indicate that displacement causes child marriage rates to substantially increase [24, 35, 36], this study uncovers evidence that child marriage may potentially be less practiced now than it used to be, as suggested by the low prevalence of child marriage among younger cohorts compared to their older counterparts. Several reasons may explain the observed findings. For one, this could be explained by general downward secular trends in age at marriage – unrelated to displacement [37]. The protracted nature of displacement of Somali refugees – evidenced by the finding that most respondents lived in Kobe camp for many years – may also indicate that vulnerability is less heightened now than in the immediate aftermath of displacement. Because several international and national organizations offer intensive social and health services in the camp, it is plausible that increased access to schooling among girls coupled with better sensitization of families to the dangers of child marriage have contributed to a shift in marriage practices. Indeed, a recent study in Dolo Odo by Sharma et al. found evidence for perceived changes in child marriage which were attributed to “NGO awareness-raising programs and Ethiopian laws prohibiting child marriage as well as increased access to education for girls.” [38] Nonetheless, the low rates can in part be due to underestimation of child marriage prevalence among 15–19-year-old girls due to right censoring as well as potential underreporting stemming from social desirability bias. That the practice was still less frequently reported among 18- and 19-year-old women who completed time at risk of child marriage compared to older women suggests that right censoring is not entirely the reason behind the low rates. Additionally, our finding that child marriage was commonly reported among older cohorts of women implies that social desirability bias is not generally that common as women tended to be forthcoming about their age at marriage.

In addition to providing information on the proportion of child marriage among adolescent girls, this study identifies several risk factors of child marriage in this context. Multivariable analysis of sociodemographic, family- and displacement-related risk factors of child marriage reveals several important patterns. Consistent with broader literature on child marriage [39–45], girls who attend school were less likely to experience child marriage even after adjusting for other variables. Explanations of the relationship between education and age at marriage are manifold, but two explanations are particularly relevant for this context. For one, girls attending school may be more likely to exercise autonomy and assert preferences around marriage compared to their counterparts who are not in school [43, 46]. Additionally, girls who are not enrolled in school may have no viable alternatives especially considering the limited opportunities for employment in this setting. Marriage may thus be deemed by parents as the only option for out-of-school girls.

In conflict settings, the link between education and child marriage has been previously demonstrated. Research undertaken among Congolese refugees in Nakivale settlement in Uganda revealed that constraints in accessing education and language barriers contributed to school drop-out which led to child marriage [23]. Similarly, research among Syrian refugees echoes findings that disruption to education occurring in the aftermath of conflict increased girls’ confinement and thus exacerbated their vulnerability to child marriage [47, 48]. These findings indicate that camp policies and programs which promote access to education and which support girls to stay in school could lead to meaningful declines in child marriage in this population. Variables associated with modernization and cultural background such as urban residence in Somalia and ethnicity were not found to be associated with child marriage - despite being shown in the literature to be important correlates of marriage [43, 49–51]. The prolonged nature of displacement in this sample and the fact that Somali refugees of different ethnicities are intermixed may have attenuated differences in behaviors stemming from ethnicity and residence of origin in Somalia.

The study also reveals that several family characteristics are associated with child marriage. A very strong association was detected between hazard of marriage and sex of the head of household. These results could imply an effect of poverty on child marriage because female-headed households may have fewer income earners and thus tend to be more vulnerable to impoverishment, thereby standing to benefit from shifting the financial burden of their daughters onto a male suitor. Few studies have found evidence for a link between sex of household head and child marriage [52, 53], but several found an impact of household poverty on children’s risk of child marriage [50, 52, 54].

We also found evidence for “sister effects.” That respondents from households with multiple girls under age 18 were less likely to marry early could imply one of two things. First, it is possible that parents seek to arrange marriages of their daughters in exact birth order, opting to marry older girls first, and thus girls with older sisters may be less vulnerable to child marriage. Indeed, Pesando and Abufhele found evidence of such an effect in India, Peru and Vietnam – although the relationship seemed to flip in Ethiopia [34]. Another possible explanation for this finding pertains to the protective effect of having sisters in the household. Evidence from sub-Saharan Africa indicates that in societies with pro-male bias, families face constraints in the financial resources they can devote to their children – specifically in relation to education [55]. This triggers rivalries for scarce resources in which parents tend to favor their male children; therefore, girls stand to gain (for example, by being retained in school) if their siblings are girls, not boys. More research is needed to illuminate the reasons behind potential ‘sister effects’ in this setting.

In terms of displacement-related characteristics, our study did not find an effect of displacement duration nor of number of times a household moved in the past year on the hazard of child marriage. Both variables are proxies for instability resulting from refugee status. That they were both not associated with child marriage can again be explained by the fact that most respondents were residing in Kobe camp for an extended period of time, and their lives have been fairly stable over the past several years. Indeed, only a few respondents reported being mobile in the past 12 months before the survey and only a few were new arrivals to the camp. We did, however, find evidence that unemployment which serves as a proxy for economic deprivation was associated with increased likelihood of child marriage. In January 2019, Ethiopia amended its existing national refugee law, providing refugees with the right to work and with expanded employment opportunities [56]. This is a welcome move that promises to afford financial protection to many families. Whether improved economic opportunities will contribute to declines in child marriage among Somali refugees is an area for future research.

Despite the observed decline in child marriage among younger girls, our research still points to persistent gaps in knowledge and attitudes about age at marriage among female adults. As illustrated, women who married their girls early tended to incorrectly identify legal age at marriage for girls and reported low marital age preference for both girls and boys. Although both Somalia and Ethiopia have enacted laws that set out the minimum age of marriage at 18 [57], inability to identify age 18 as the minimum age at marriage was common among female adults. This points to the need to raise awareness among refugee women, girls, boys and community members at large to the legal minimum age of marriage and the harms of early marriage more generally. Adult women were also more likely to identify boys as decision-makers in marriage as compared to girls, illustrating the need to sensitize parents to the importance of marital choice for girls as well as empowering girls to assert their preferences. Addressing religious views propelling this practice is also necessary given the apparent salience of religious traditions as a driver of child marriage in this context. Working with religious leaders may be one crucial way to curb this practice, especially since religious ceremonies are widespread and religious leaders play a central role in officiating marriages in this setting.

There is also pressing need to address gaps in knowledge and practices around contraceptives among married adolescent girls. Like other studies in different settings, our findings indicate that married adolescent girls rarely use contraception and are thus unable to exercise reproductive control [58–60]. In this population, there is a dearth of studies investigating knowledge, attitudes and practices around contraceptive use among married adolescent girls, so more in-depth data is needed to understand exact reasons behind non-use and how best to support married adolescent girls and boys with contraceptive information, counseling and methods. The finding that many adolescent girls were in marriages with large spousal age difference – presumably leading to large power differentials – also raises concerns that girls may be vulnerable to abuse and domestic violence. Taken together, these findings highlight the need to focus programs not only on prevention of child marriage but also on mitigating its negative consequences through targeting interventions to married adolescent girls. Evidence indicates that multi-layered interventions that target girls and boys, their families, peers, community leaders and the health system have considerable potential to delay pregnancy and improve contraceptive uptake and pregnancy care in this group [61].

There are several limitations to our findings. For one, the sample of married women was small and thus many of our confidence intervals are wide. This is because our sample was not powered to test specific hypotheses and was instead calculated with the intention of estimating a precise estimate of prevalence of child marriage. Notwithstanding the small sample size, this study is one of the first studies dedicated to quantitatively measuring child marriage among Somali refugees in Ethiopia and findings have several important implications for sexual and reproductive health as well as gender programming. Moreover, the data came from a cross-sectional survey which precludes drawing causal inference. Because we rely on retrospective reporting of age of marriage, our findings are subject to recall and social desirability bias – although the former is unlikely among younger women given that less time had elapsed between the survey and the event of interest. Survey respondents may have also underreported their marital age out of fear of repercussions given that child marriage is illegal. Misreporting was reduced by daily quality checks undertaken by the research coordinator in which discrepancies in reported age of marriage were investigated and corrected. Another limitation is that our models lacked a validated measurement of household poverty or socioeconomic status (SES). Because it is difficult to measure SES in humanitarian settings, we tried to use proxy indicators such as education, work status of the HH, and residence in Somalia. However, these may not fully reflect current SES and there is thus possibility that our findings are affected by residual confounding. Future research should examine the impact of direct measures of SES on marriage practices in humanitarian settings.

## Conclusions

The findings of the study illustrate the ways in which humanitarian crisis and protracted displacement impact risk and drivers of child marriage. They also offer insights into how to protect girls from child marriage and facilitate their healthy transitions into adulthood. In addition to targeting girls at risk of marriage, programs should aim to mitigate the risks posed by marriage to adolescent girls’ health and wellbeing.

## Supplementary Information

**Additional file 1:.**

**Additional file 2:.**

## Abbreviations

JHU: Johns Hopkins University
MICS: Multiple Indicator Cluster Survey
SES: Socioeconomic status
CIs: Confidence intervals

## Footnotes

1 When asked about their relationship to the head of household, respondents were given several choices including “spouse of head of household”, or “head of household”. Households in which respondents identified as heads of household were classified as female-headed. However, it is important to note that UNHCR designates the role of head of household for aid distributions, and in many cases the woman is chosen as the head of household even if a male is present because women tend to be less mobile. It is thus possible that some respondents stated that they were the heads of household because of the UHCR designation in spite of the presence of a male in the household.
