# Supplementary material for: Child marriage among Somali refugees in Ethiopia: a cross sectional survey of adolescent girls and adult women
Source: BMC Public Health. 2021 Jun 2;21:1051. doi: 10.1186/s12889-021-11080-5 (PMC8173891; doi:10.1186/s12889-021-11080-5)
Supplement: Supplementary file 1 — Additional file 1:. [file 12889_2021_11080_MOESM1_ESM.docx]

**SURVEY INSTRUMENT – Girls/Women 10-19**

**Early Marriage in Humanitarian Contexts**

| **SECTION 1: INTERVIEW CONTEXT**  ***[This first set of questions is to be filled out by the interviewer with the respondent’s help when needed]*** | | | | |
| --- | --- | --- | --- | --- |
| **AA01** | Location ID | | [__\|__] | |
| **AA02** | Cluster ID | | [__\|__] | |
| **AA03** | Household ID | | [__\|__] | |
| **AA04** | Respondent ID number | | [__\|__\|__] | |
| **AA05** | Interviewer’s name and ID number | | _______________ [__\|__] | |
| **AA06** | Date of interview | | [__\|__][__\|__] 2019  DD MM | |
| **AA07** | Record the time | | [__\|__][__\|__]  HH MM | |
| **AA08** | *Was anyone else present during this interview?*  ***[Interviews should be conducted individually with each adolescent. If it is not possible to obtain this level of privacy and another individual is insistent upon being present—please circle “yes” for this question]*** | | No……………....0  Yes……………...1 | 🡪 skip to AA10 |
| **AA09** | Who was present? | | Child 10 years or younger………………..0  Female older than 10 years…………..….1  Male older than 10 years…………………2 | |
| **AA10** | How long have you lived in [*current location*]?  *All years should be multiplied by 12 to get the correct number (1 years = 12 months, 1.5 years = 18 months).* | [__\|__] | Months  (fill 88 if don’t know, fill 99 if refused response) | |

| **SECTION 2: ADOLESCENT SOCIODEMOGRAPHIC INFORMATION AND RISK FACTORS** | | | | |
| --- | --- | --- | --- | --- |
| **AB01** | How old are you today?  *Probe: How old did you turn at your last birthday?* | [__\|__] Age (years) | |  |
| **AB02** | What is your marital status? | Single………………………………………………..……………………….0  Engaged……………………………………………………………………..1  Married………………………………….…………..……………………….2  Widowed……………………………………………..………………..…….3  Divorced …………………………………………..…………………..…….4  Separated……………………………………………..…………………….5Don’t know……………………………………………………………….88  Refused response………………………………………………………99 | | 🡪 skip to AB05  🡪 skip to AB04 |
| **AB03** | At what age did you first marry? | [__\|__] | Years  (fill 88 if don’t know, fill 99 if refused response) |  |
| **AB04** | At what age did you become engaged? | [__\|__] | Years  (fill 88 if don’t know, fill 99 if refused response) |  |
| **AB05** | Do you currently work outside the home? | No…………………………………………………..…………………….....0  Yes…………………………………………………..……………………...1 | | 🡪 skip to AB07 |
| **AB06** | What kind of work do you do? | Meat/dairy production………………………………………...…………...0  Hairdresser salon……..…………….…………………..………………...1  Sewing shop…………………………………………………………..…...2  Clothing/accessory shop………………………………………………….3  Domestic labor……………………………………………………………..4  Agriculture/Farming………………………………………………………..5  Fishing………………………………………………………………………6  Masonry…………………………………………………………………….7  Other (specify)___________________________________________  Don’t know……………………………………………………………….88  Refused response………………………………………………………99 | |  |
| **AB07** | Are you paid cash, in-kind, or not at all? | Not at all…………………………………………………..………………..0  Cash………………………………………………..……………………....1  Kind………………………………………………..…………………….....2  Don’t know……………………………………………………………….88  Refused response………………………………………………………99 | |  |
| **AB08** | The next question asks about difficulties doing certain activities because of a physical or mental disability. Do you have moderate to severe difficulties? | No…………………………………………………..……………………….0  Yes…………………………………………………..……………………...1  Don’t know……………………………………………………………….88  Refused response………………………………………………………99 | | 🡪 skip to AB10 |
| **AB09** | What kind of disability do you experience? | Seeing, even if wearing glasses………………………………………..…0  Hearing, even if using a hearing aid……………………………………...1  Walking or climbing steps……………………………………………….…2  Remembering or concentrating…………………………………………...3  Caring for themselves, such as washing all over or dressing……….…4  Communicating, either expressing themselves or being understood…5  None of the above…………………………………………………………..6  Don’t know………………………………………………………...……….88  Refused response………………………………………………….……..99 | |  |
| **AB10** | In the household you were born into, did you have any of the following: Radio? Television? Goat? | None………………………………………………………………………...0  Radio…………..…………………………………..…………………….....1  Television…………………………………………………………………..2  Goat…………………………………………………………………………3  Don’t know………………………………………………………...……….88  Refused response………………………………………………….……..99 | |  |
| **AB10** | Have either of your parents passed away? | No…………………………………………………..……………………….0  Yes…………………………………………………..………………………1  Don’t know………………………………………………………...……….88  Refused response………………………………………………….……..99 | | 🡪 skip to AB12 |
| **AB11** | Which of your parents passed away? | Mother………………………………………………….…………...………0  Father…………………………………………………………………..…...1  Both……………………………………………………………………..…..2  Don’t know………………………………………………………...……….88  Refused response………………………………………………….……..99 | |  |
| **AB12** | Are you currently in school? | No…………………………………………………..……………………….0  Yes…………………………………………………..……………………...1  Don’t know………………………………………………………...……….88  Refused response………………………………………………….……..99 | | 🡪 skip to AB15 |
| **AB13** | Who made the decision you should not go to school? | Me…………………………………………………………………………….0  My father or my husband ……….………………………………………....1  My mother or mother-in-law …………………………………………….…2  Other (specify) _________________________________________  Don’t know………………………………………………………………....88  Refused response…………………………………………………………99 | |  |
| **AB14** | What is the **primary reason** you/they made this decision? | Cost (school fees/transportation)…..……………………...……………0  Distance to school is too far…………………………………………...…1  Lack of safety in transit to school………………………………………..2  Language of instruction not appropriate..…………………………..…..3  No certificate offered for completion…………………………………….4  Lack of safety/discrimination in schools..……………………………….5  I am needed at home……………………………………………………...6  Other (specify) ___________________________________________  Don’t know………………………………………………………………....88  Refused response…………………………………………………………99 | |  |
| **AB15** | These next questions are about having children of your own. Have you ever given birth? | No…………………………………………………..……………………….0  Yes…………………………………………………..………………………1  Don’t know………………………………………………………………....88  Refused response…………………………………………………………99 | | 🡪 skip to Section 3 |
| **AB16** | At what age did you first give birth? | [__\|__] | Years  (fill 88 if don’t know, fill 99 if refused response) |  |
| **AB17** | How many live children do you have? | [__\|__] | Years  (fill 88 if don’t know, fill 99 if refused response) |  |
| **AB18** | Sometimes it happens that children die. It may be painful to talk about and I’m sorry to ask about painful memories, but it is important to get correct information. Have you given birth to a baby who was born alive but later died?    ***If No, probe:*** Any baby who cried or showed signs of life, but later died? | No…………………………………………………..……………………….0  Yes…………………………………………………..……………………...1  Don’t know………………………………………………………………....88  Refused response…………………………………………………………99 | | 🡪 skip to AB21 |
| **AB19** | How many girls died? | [__\|__] | Total number of girls who died  (fill 88 if don’t know, fill 99 if refused response) |  |
| **AB20** | How many boys died? | [__\|__] | Total number of boys who died  (fill 88 if don’t know, fill 99 if refused response) |  |
| **AB21** | To be sure I understand correctly, how many births have you had?  ***Check by summing answers AB23, AB25, and AB26 to confirm AB27 is the accurate number.*** | [__\|__] | Total number of births  (fill 88 if don’t know, fill 99 if refused response) |  |
| **AB22** | Now I would like to ask you about your last pregnancy. These questions might be sensitive for some people, but all responses are anonymous and confidential. If you have any questions or concerns, let me know.  When you became pregnant, did you want to have another child at all? Did you want to wait until later or did you not want to have another child? | Not at all…………………………………………………..……..…………..0  At that time…………………………………………………………………..1  Later………………………………………………..…………….…………..2  Don’t know………………………………………………………………...88  Refused response………………………………………………………..99 | |  |
| **AB23** | Have you ever used a contraceptive method? | No…………………………………………………..……………….………..0  Yes…………………………………………………..……………………….1  Don’t know………………………………………………………………….88  Refused response…………………………………………………………99 | | 🡪 skip to Secion 3 |
| **AB24** | What contraceptive method did you use? | Male condom………………………………………..……………….………0  Female condom………………………………………..……………….…...1  Male sterilization………………………………………..……………….…..2  Female sterilization………………………………………..……………….3  IUD………………………………………..……………….………………….4  Implant………………………………………..……………….……………..5  Pill………………………………………..……………….…………………..6  Diaphragm………………………………………..……………….…………7  Other (specify)________________________________________  Don’t know………………………………………………………………….88  Refused response…………………………………………………………99 | |  |

| **SECTION 3: HOUSEHOLD OF ORIGIN CHARACTERISTICS** | | | | | |
| --- | --- | --- | --- | --- | --- |
| **AC01** | Were you born into this household? | No…………………………………………………..……………………….0  Yes…………………………………………………..……………………...1  Don’t know………………………………………………………...……….88  Refused response………………………………………………….……..99 | | | 🡪 skip to Section 4 |
| **AC02** | Did you marry into this household? | No…………………………………………………..……………………….0  Yes…………………………………………………..……………………...1  Don’t know………………………………………………………...……….88  Refused response………………………………………………….……..99 | | |  |
| **AC03** | When you were growing up, how many people in your household of origin were under the age of 18? | [__\|__] | People  (fill 88 if don’t know; fill 99 if refused response) | |  |
| **AC04** | What is the highest level and grade or year of school that your father, or male he*ad of your household of origin* completed? | Preschool…………………….………0  Primary……………………………….1  Secondary…………………………...2  Higher…………………………….….3  Don’t know………………………….88  Refused response…………………99 | | [__\|__] Grade  (fill 88 if don’t know, fill 99 if refused response) |  |
| **AC05** | What was the most recent occupation of your he*ad of household* of origin prior to displacement from Somalia? | Domestic work ………………………….…….......……………………....0  Religious or community leader …………………………………..…..….1  Teacher or NGO employee …….…………………………..………...….2  Medical or healthcare……………………………………..…………...….3  Trader or Merchant………………………………………………………..4  Agriculture/Farming………………………………………………………..5  Fishing………………………………………………………………………6  Masonry…………………………………………………………………….7  Unemployed………………………………………………………………..8  Other (specify)___________________________________________  Don’t know………………………………………………………...……….88  Refused response………………………………………………….……..99 | | |  |
| **AC06** | Is the head of your household of origin working currently? | No…………………………………………………..……………………..0  Yes…………………………………………………..…………………….1  Don’t know………………………………………………………...……….88  Refused response………………………………………………….……..99 | | | 🡪 skip to AC08 |
| **AC07** | What type of work does the head of your household of origin currently do? | Domestic work ………………………….…….......……………………....0  Religious or community leader …………………………………..…..….1  Teacher or NGO employee …….…………………………..………...….2  Medical or healthcare……………………………………..…………...….3  Trader or Merchant………………………………………………………..4  Agriculture/Farming………………………………………………………..5  Fishing………………………………………………………………………6  Masonry…………………………………………………………………….7  Other (specify)___________________________________________  Don’t know………………………………………………………...……….88  Refused response………………………………………………….……..99 | | |  |
| **AC08** | Where is the head of your household of origin from originally in Somalia? | Bay region..……………………………..………………………………….0  Bakool region.………………………………………………..…………….1  Geda regon.………………………………………………..……………….2  Shabelle region.………………………………………………..…………..3  Other (specify)____________________________________________  Don’t know………………………………………………………...……….88  Refused response………………………………………………….……..99 | | |  |
| **AC09** | Would you describe the head of your household of origin’s place of origin as urban or rural? | Urban…………………………..………………………….…….………..….0  Rural………..………………………………………………………………..1  Don’t know………………………………………………………...……….88  Refused response………………………………………………….……..99 | | |  |
| **AC10** | Was there a female head of household consistently present in your household of origin? | No…………………………………………………..……………………….0  Yes…………………………………………………..……………………...1  Don’t know………………………………………………………………...88  Refused response……………………………………………………..…99 | | | 🡪 skip to AC13 |
| **AC11** | Did your mother, or *female head of household* work after displacement? | No…………………………………………………..……………………..0  Yes…………………………………………………..…………………….1  Don’t know………………………………………………………………...88  Refused response……………………………………………………..…99 | | | 🡪 skip to AC13 |
| **AC12** | What kind of work did she (mother/*female head of household*) do in Ethiopia? | Domestic work ………………………….…….......……………………....0  Religious or community leader …………………………………..…..….1  Teacher or NGO employee …….…………………………..………...….2  Medical or healthcare……………………………………..…………...….3  Trader or Merchant………………………………………………………..4  Agriculture………………………………………………………………….5  Other (specify)___________________________________________  Don’t know………………………………………………………………...88  Refused response……………………………………………………..…99 | | |  |
| **AC13** | How many times did your household move within the past 12 months? | [__\|__] | Times  (fill 88 if don’t know, fill 99 if refused response) | |  |

| **SECTION 4: MARRIAGE PERCEPTIONS** | | | | | |
| --- | --- | --- | --- | --- | --- |
| ***Read the folllowing to the participant:*** *“*Now, I am going to read you seven statements about marriage. To respond to each statement, I would like you to please indicate if you ‘strongly agree’, ‘agree’, ‘feel neutral’, ‘disagree’, or ‘strongly disagree’ with each statement. If you are confused at any point, please let me know.” | | | | |  |
| **AD01** | ***Read to participant:*** *“*Marriage is important for maintaining family honor and reputation.*”* | Strongly agree……………………………………………………….………..0  Agree……………………………………………………………..……………1  Disagree……………………………………………………………………….2  Strongly disagree……………………………………………………………..3  Don’t know…………………………………………………………………...88  Refused response………………………………………………………..…99 | | |  |
| **AD02** | ***Read to participant:*** *“*The age at which I marry, is a decision that my parents or relatives should make for me.” | Strongly agree……………………………………………………….………..0  Agree……………………………………………………………..……………1  Disagree……………………………………………………………………….2  Strongly disagree……………………………………………………………..3  Don’t know…………………………………………………………………...88  Refused response………………………………………………………..…99 | | |  |
| **AD03** | ***Read to participant:*** *“*Girls should complete school before they marry.” | Strongly agree……………………………………………………….………..0  Agree……………………………………………………………..……………1  Disagree……………………………………………………………………….2  Strongly disagree……………………………………………………………..3  Don’t know…………………………………………………………………...88  Refused response………………………………………………………..…99 | | |  |
| **AD04** | ***Read to participant:*** *“*Marriage would help me to overcome some of the challenges I face.” | Strongly agree……………………………………………………….………..0  Agree……………………………………………………………..……………1  Disagree……………………………………………………………………….2  Strongly disagree……………………………………………………………..3  Don’t know…………………………………………………………………...88  Refused response………………………………………………………..…99 | | |  |
| **AD05** | ***Read to participant:*** *“*Girls should not attend school once they are married.” | Strongly agree……………………………………………………….………..0  Agree……………………………………………………………..……………1  Disagree……………………………………………………………………….2  Strongly disagree……………………………………………………………..3  Don’t know…………………………………………………………………...88  Refused response………………………………………………………..…99 | | |  |
| **AD06** | ***IF PARTICIPANT IS MARRIED—Read to participant:*** *“*I was prepared to become a wife at the time I married.”  ***IF PARTICIPANT IS UNMARRIED—Read to participant:*** *“*I feel prepared to marry.” | Strongly agree……………………………………………………….………..0  Agree……………………………………………………………..……………1  Disagree……………………………………………………………………….2  Strongly disagree……………………………………………………………..3  Don’t know…………………………………………………………………...88  Refused response………………………………………………………..…99 | | |  |
| **AD07** | ***Read to participant:*** “I will be able to make more decisions about my life when I become married.” | Strongly agree……………………………………………………….………..0  Agree……………………………………………………………..……………1  Disagree……………………………………………………………………….2  Strongly disagree……………………………………………………………..3  Don’t know…………………………………………………………………...88  Refused response………………………………………………………..…99 | | |  |
| **AD08** | At what age do you think people in your community should marry? | **FEMALES** | | **MALES** |  |
|  |  | [__\|__] Age (years)  (fill 88 if don’t know; fill 99 if refused response) | | [__\|__] Age (years)  (fill 88 if don’t know; fill 99 if refused response) |  |
| **AD09** | In your community, what primarily influences decisions about the age people become married? | Religion…………………………………………….……………….……….0  Family honor……………………………………….………………………..1  Family tradition…………………………………….………………………..2  Money and/or resources……………………………….…………………..3  War and/or conflict…………………………………………….……………4  Displacement………………………………………………….…………….5  Other (specify) ___________________________________________  Don’t know…………………………………………………………………...88  Refused response………………………………………………………..…99 | | |  |
| **AD10** | Is this the same factor that influenced decisions about the age people became married prior to displacement? | No………………………………………………………………………..…..0  Yes…………………………………………………………………………..1  Don’t know…………………………………………………………………...88  Refused response………………………………………………………..…99 | | | 🡪 skip to AD12 |
| **AD11** | In your community, what primarily influenced decisions about the age people become married, prior to displacement? | Religion…………………………………………….……………….……….0  Family honor……………………………………….………………………..1  Family tradition…………………………………….………………………..2  Money and/or resources……………………………….…………………..3  War and/or conflict…………………………………………….……………4  Displacement………………………………………………….…………….5  Other (specify) ___________________________________________  Don’t know…………………………………………………………………...88  Refused response………………………………………………………..…99 | | |  |
| **AD12** | In your community today, who most significantly influences the age at which a boy will marry?  ***[Circle all that are mentioned]*** | The boy……………………………………………………….…….…………0  The girl…………………………………………………………….………….1  The boy’s parents………………………………………………….………...2  The girl’s parents………………………………….………………………....3  The boy’s relatives……………………..………………………………..…..4  The girl’s relatives…………………………………………………..……….5  Other (specify)_____________________________________________  Don’t know…………………………………………………………………...88  Refused response………………………………………………………..…99 | | |  |
| **AD13** | In your community today, who most significantly influences the age at which a girl will marry?  ***[Circle all that are mentioned]*** | The boy……………………………………………………….…….……….…0  The girl…………………………………………………………….………..….1  The boy’s parents………………………………………………….…….…...2  The girl’s parents………………………………….…………………….…....3  The boy’s relatives……………………..………………………………..……4  The girl’s relatives…………………………………………………..….……..5  Other (specify)_____________________________________________  Don’t know…………………………………………………………………...88  Refused response………………………………………………………..…99 | | |  |
| **AD14** | In your community, is there typically an age difference between the bride and the groom? | No……………………………………………………………………………..0  Yes………………………………………………………………………..…..1  Don’t know…………………………………………………………………...88  Refused response………………………………………………………..…99 | | | 🡪 skip to AD16 |
| **AD15** | In your community, what is typically the age difference between the bride and the groom when they get married? | Man is older than bride by less than 5 years………….…………………0  Man is older than the bride by 5-10 years………………………….……..1  Man is older than the bride by 10 years or more…………………………2  Don’t know…………………………………………………………………...88  Refused response………………………………………………………..…99 | | |  |
| **AD16** | What is the legal, minimum age of marriage for girls? | [__\|__] | Years  (fill 99 if don’t know) | |  |
| **AD17** | What is the legal, minimum age for marriage for boys? | [__\|__] | Years  (fill 99 if don’t know) | |  |

| **SECTION 5: BARRIERS TO PROGRAM ENGAGEMENT** | | | | |
| --- | --- | --- | --- | --- |
| **AE01** | Who **primarily** decides what activities are appropriate for you to do outside of the home? | Yourself……………………………………………………….…….…….……0  You in collaobration with another family member …………….……….….1  Your husband …………………………………………………………………2  Mother/ female head of household……………………………….…….......3  Father/ male head of household………………………………………….....4  Other male relative(s)…………………..………………………………..…..5  Other female relative(s) …...………………………………………..……….6  Don’t know…………………………………………………………………...88  Refused response………………………………………………………..…99 | |  |
| **AE02** | What activities are important for you to participate in?  ***[Read options aloud to participant and circle all that are mentioned]*** | Education…………………………………………………….…….…….……0  Livelihoods………………………………………………….…….…….……..1  Psychosocial……………………………………………………….…….……2  Life skills……………………………………………………….…….…….…..3  Peer programs/mentorship……………………………………….………….4  Don’t know…………………………………………………………………...88  Refused response………………………………………………………..…99 | |  |
| **AE03** | How much free time do you have each day? We would like to know the number of hours during which you are not in school or doing domestic work. | [__\|__] | Hours  (fill 99 if don’t know) |  |
| **AE04** | During what time of day does this free time occur? | Morning………………………………………………………………..………0  Afternoon………………………………………………………………..……1  Evening………………………………………………………………..………2  Don’t know………………………………………………………………..….88  Refused response…………………………………………………,,………99 | |  |
| **AE05** | When programs are established in this community, for adolescent girls, like yourself—what is the greatest challenge you face in attending?  ***FHH = Female Head of Household***  ***HH = Head of Household***  ***[Circle all that are mentioned]*** | I am not interested…………….…………………………………………...….0  Mother/ FHH is not comfortable…………….…………………………….….1  Father/ HH is not comfortable.……………………………..…….……….….2  I have other responsibilities……………………………..…….……….…….3  School……………………………..…….…………………………….…….….4  Programs are too far away……………………………..…….……….……...5  Other (specify)______________________________________________  Don’t know………………………………………………………………..….88  Refused response…………………………………………………,,………99 | |  |

| **SECTION 6: HEALTH AND SERVICE KNOWLEDGE** | | | |
| --- | --- | --- | --- |
| ***These next questions are about women and girl’s health. We will ask you about your awareness of different health issues. Do you feel comfortable anwering these questions?*** | | No………………………………………………………………………..…..0  Yes…………………………………………………………………………..1 | 🡪 skip to Section 6 |
| ***Read the folllowing to the participant:*** *“*Now, I am going to read you eight statements about health. To respond to each statement, I would like you to please indicate if you agree by saying “yes” or “no.” Remember all answers are anonymous and you do not have to answer every question. If you are confused at any point, please let me know.” | | |  |
| **AF01** | ***Read to participant:*** *“*I feel comfortable with the changes that occur in my body as I shift from being a child to an adult.” | No……………………………………………………………………………..0  Yes………………………………………………………………………..…..1  Don’t know…………………………………………………………………...88  Refused response………………………………………………………..…99 |  |
| **AF02** | ***Read to participant:*** *“*I have received information about how to become pregnant.” | No……………………………………………………………………………..0  Yes………………………………………………………………………..…..1  Don’t know…………………………………………………………………...88  Refused response………………………………………………………..…99 |  |
| **AF03** | ***Read to participant:*** *“*I know how to keep my body healthy when I am pregnant.” | No……………………………………………………………………………..0  Yes………………………………………………………………………..…..1  Don’t know…………………………………………………………………...88  Refused response………………………………………………………..…99 |  |
| **AF04** | ***Read to participant:*** *“*I am aware of danger signs during pregnancy, which would require me to go to hospital.” | No……………………………………………………………………………..0  Yes………………………………………………………………………..…..1  Don’t know…………………………………………………………………...88  Refused response………………………………………………………..…99 |  |
| **AF05** | ***Read to participant:*** *“*It can be harmful to a woman’s body to have children when she is too young.” | No……………………………………………………………………………..0  Yes………………………………………………………………………..…..1  Don’t know…………………………………………………………………...88  Refused response………………………………………………………..…99 |  |
| **AF06** | ***Read to participant:*** *“*It can be harmful to a woman’s body to give birth to children too close together.” | No……………………………………………………………………………..0  Yes………………………………………………………………………..…..1  Don’t know…………………………………………………………………...88  Refused response………………………………………………………..…99 |  |
| **AF07** | ***Read to participant:*** *“*There way ways to prevent or delay pregnancy.” | No……………………………………………………………………………..0  Yes………………………………………………………………………..…..1  Don’t know…………………………………………………………………...88  Refused response………………………………………………………..…99 |  |
| **AF08** | ***Read to participant:*** *“*I have someone I trust, who I can get information about my health from.” | No……………………………………………………………………………..0  Yes………………………………………………………………………..…..1  Don’t know…………………………………………………………………...88  Refused response………………………………………………………..…99 |  |
| **AF09** | Have you ever been to the health clinic in Ethiopia? | No………………………………………………………………………..……..0  Yes………………………………………………………………………....…..1  Don’t know…………………………………………………………………...88  Refused response………………………………………………………..…99 |  |
| **AF10** | Did you feel comfortable with the services you received at the health clinic in Ethiopia? | No………………………………………………………………………..……..0  Yes………………………………………………………………………....…..1  Don’t know…………………………………………………………………...88  Refused response………………………………………………………..…99 |  |
| **AF11** | Why were you not comfortable with the services you received at the health clinic?  ***[Check all that are mentioned]*** | Providers were not respectful……………………………………………….0  Lack of confidence in providers…………………………………………….1  Did not feel safe……………………………………………………………….2  Long wait times………………………………………………………………..3  Services provided are ineffective……………………………………………4  Services are too expensive………………………………………………….5  Unsanitary/poor physical environment……………………………………..6  Other (specify)____________________________________________  Don’t know……………………………………………………………………88  Refused response…………………………………………………………..99 |  |

| **SECTION 7: INTERVENTION EXPOSURE** | | | | |
| --- | --- | --- | --- | --- |
| **AG01** | Have you recieved any information or education about the effects of child marriage on girls? | No………………………………………………………………………………..0  Yes………………………………………………………………………..…..…1  Don’t know……………………………………………………………………88  Refused response…………………………………………………………..99 | | 🡪 skip to AG04 |
| **AG02** | What format did you receive this information?  ***[Circle all that are mentioned]*** | Pamphlet………………………………………………………………………...0  Community meeting……………...…………………………………………….1  Radio…………………………………………………………………………….2  Other (specify)____________________________________________  Don’t know……………………………………………………………………88  Refused response…………………………………………………………..99 | |  |
| **AG03** | Do you know which organization provided this information to you? | No………………………………………………………………………………..0  Yes…………………………………………………………………………..…..1  Please specify the name of the organization: ___________________  Refused response…………………………………………………………..99 | |  |
| **AG04** | Have you ever participated in educational programs for girls outside of school? | No……………………………………………………………………………..…0  Yes………………………………………………………………………..……..1  Don’t know……………………………………………………………………88  Refused response…………………………………………………………..99 | | 🡪 skip to AG06 |
| **AG05** | What types of educational programs have you participated in?  ***[Circle all that are mentioned]*** | Life skills training………………………………………………….……………0  Mentorship program…………………………………………………………....1  Community presentation………………………………………………..…..…2  Other (specify): ____________________________________________  Don’t know……………………………………………………………………88  Refused response…………………………………………………………..99 | |  |
| **AG06** | Have you ever received financial support—money or resources—to attend school? | No……………………………………………………………………………..…0  Yes………………………………………………………………………..……..1  Don’t know……………………………………………………………………88  Refused response…………………………………………………………..99 | | 🡪 skip to AG08 |
| **AG07** | Do you know which organization provided this information to you? | No………………………………………………………………………………..0  Yes………………………………………………………………………..……..1  Please specify the name of the organization: __________________  Refused response…………………………………………………………..99 | |  |
| **AG08** | How many times in the past month have you attended the Women and Girls Center? | [__\|__] | Times  (fill 88 if don’t know, fill 99 if refused response) |  |
| **AG09** | Have you ever been told that marriage of children before the legal, minimum age is harmful? | No………………………………………………………………………………..0  Yes………………………………………………………………………..……..1  Don’t know……………………………………………………………………88  Refused response…………………………………………………………..99 | |  |
| ***Read the following to the* participant:** We are done with the survey. Thank you for your time today. | | | |  |
