# Supplementary material for: Child marriage among Somali refugees in Ethiopia: a cross sectional survey of adolescent girls and adult women
Source: BMC Public Health. 2021 Jun 2;21:1051. doi: 10.1186/s12889-021-11080-5 (PMC8173891; doi:10.1186/s12889-021-11080-5)
Supplement: Supplementary file 2 — Additional file 2:. [file 12889_2021_11080_MOESM2_ESM.docx]

**SURVEY INSTRUMENT – Female Head of Household**

**Early Marriage in Humanitarian Contexts**

| **SECTION 1: INTERVIEW CONTEXT**  **[This first set of questions is to be filled out by the interviewer with the respondent’s help when needed]** | | | | |
| --- | --- | --- | --- | --- |
| **FA01** | Location ID | | [__\|__] | |
| **FA02** | Cluster ID | | [__\|__] | |
| **FA03** | Household ID | | [__\|__] | |
| **FA04** | Respondent ID number | | [__\|__\|__] | |
| **FA05** | Interviewer’s name and ID number | | _______________[__\|__] | |
| **FA06** | Date of interview | | [__\|__][__\|__] 2019  DD MM | |
| *Ask to speak with the female head of household; if unavailable ask to speak with eldest female, 18 years of age or older, who has lived in household for more than one year. If unavailable, ask to speak with a married female aged 15 or older. If all are unavailable for interview, revisit household a maximum of three total visits (initial visit + two additional attempts).* | | | | |
| **FA07** | Person interviewed  *If respondent is “other woman over 18 yrs” of “other woman below 18 yrs” specify relationship to HH. E.g. if respondent is the daughter of the female head of household, write “daughter” on the line.* | | Female head of household………………0  Other woman over 18yrs…………………1  Other woman below 18yrs……………….2  (relationship to HH)__________________ | |
| **FA08** | Record the time interview began | | [__\|__][__\|__]  HH MM | |
| **FA09** | Was anyone else present during this interview? | | No.……………….0  Yes……………….1 | 🡪 skip to FA11 |
| **FA10** | Who was present? | | Child 10 years or younger………………..0  Female older than 10 years…………..….1  Male older than 10 years…………………2 | |
| **FA11** | How long have you lived in [*current location*]?  *All years should be multiplied by 12 to get the correct number (1 year = 12 months, 1.5 years = 18 months)* | [__\|__] | Months  (fill 88 if don’t know) | |

| **SECTION 2: LIST OF HOUSEHOLD MEMBERS**  *First complete, on a separate paper form, a list of the names for all individuals who have been members of this household for more than one month, within the past year, including yourself. The list will be used to guide the collection of data on household members, but will be destroyed after the interview. In the order of the household list, proceed with FB02 and FB03 vertically. Once FB02 and FB03 are complete for all members, make sure to probe for additional members: Those with disabilities, those that are not currently at home, or any infants, small children or others who may have recently moved out (such as girls who may have been recently married). Then, ask questions FB04-FB05 for each member one at a time.* | | | | | |
| --- | --- | --- | --- | --- | --- |
| **FB01**.  *Line*  *no.* | **FB02**.  What is the relationship of (*name*) to the head of household? | **FB03**.  Is (*name*) male or female?  1 = Male  2 = Female | | **FB04**.  How old is (*name*)?  *Record in completed years.*  *If age is 95 or above, record ‘95’.* | **FB05.**  Is (*name)* married?  0 = No  1 = Yes  88 = Don’t know  99 = Refused response |
| Line | Relation* | M | F | Age | No Yes |
| 01 | (head of household) | 1 | 2 | __ __ | 0 1 |
| 02 | ___ ___ | 1 | 2 | __ __ | 0 1 |
| 03 | ___ ___ | 1 | 2 | __ __ | 0 1 |
| 04 | ___ ___ | 1 | 2 | __ __ | 0 1 |
| 05 | ___ ___ | 1 | 2 | __ __ | 0 1 |
| 06 | ___ ___ | 1 | 2 | __ __ | 0 1 |
| 07 | ___ ___ | 1 | 2 | __ __ | 0 1 |
| 08 | ___ ___ | 1 | 2 | __ __ | 0 1 |
| 09 | ___ ___ | 1 | 2 | __ __ | 0 1 |
| 10 | ___ ___ | 1 | 2 | __ __ | 0 1 |
| 11 | ___ ___ | 1 | 2 | __ __ | 0 1 |
| 12 | ___ ___ | 1 | 2 | __ __ | 0 1 |
| * *Codes for* ***FB03****: Relationship to head of household:* | 01 Head  02 Spouse / Partner  03 Son / Daughter  04 Son-in-law / Daughter-In-Law | 05 Grandchild  06 Parent  07 Parent-In-Law  08 Brother / Sister  09 Brother-in-law / Sister-In-Law | | 10 Uncle/Aunt  11 Niece / Nephew  12 Other relative  13 Adopted / Foster / Stepchild | 14 Servant (Live-in)  96 Other (Not related)  99 DON’T KNOW |

| **SECTION 3: LIST OF ADOLESCENT HOUSEHOLD MEMBERS**  *Identify all individuals from Section 2 roster who are male or female aged 10-24, including yourself as appropriate. Transfer line number and name from Section 2 for all HH members meeting those criteria. Then complete FC02-FC12 for each adolescent.* | | | | | | | | | | |
| --- | --- | --- | --- | --- | --- | --- | --- | --- | --- | --- |
| **FC01.**  Line number from Section 1 | **FC02.**  Has (name) ever attended school?  0 = No  1 = Yes  88 = DK  99 = RR  ***No 🡪 skip to FC05*** | **FC03.**  What was the highest level and grade or year of school (name) attended?  1 = Preschool  2 = Primary  3 = Secondary  4 = Higher  88 = DK  99 = RR | **FC04**.  How many times has he/she been married?  0 = None  1 = Once  2 = More than once  88 = DK  99 = RR  ***0 🡪 skip rest*** | **FC05**.  How old was she/he when she/he married her/his first spouse?  88 = DK  99 = RR | **FC06**.  Was this marriage before or after discplacement?  0 = Before  1 = After  88 = DK  99 = RR | **FC07**.  Is (name from roster) currently married or living with someone as if married?  0 = No  1 = Yes, married  2 = Yes, living with partner  88 = DK  99 = RR  No 🡪 skip rest | **FC0**8  How old is her spouse?  88 = DK  99 = RR | **FC09**.  Besides (Name from roster), does (her husband have any other wives or partners or does he live with other women as if married?  0 = No  1 = Yes  88 = DK  99 = RR | **FC10.**  Was (name from roster) directly involved in the choice of her husband/  partner?  0 = No  1 = Yes  88 = DK  99 = RR | **FC11.**  Was this marriage a civil, religious, traditional or other type of marriage?  0 = Civil  1 = Religious  2 = traditional  3 = Other  88 = DK  99 = RR |
| Line # | No Yes | Level |  | Age  (years) | Age  (years) | No/Yes | Age | No Yes | No Yes | Type |
|  | ____ | ____ | ____ | __ __ | ____ | ____ | ___ ___ | ____ | ____ | ____ |
|  | ____ | ____ | ____ | __ __ | ____ | ____ | ___ ___ | ____ | ____ | ____ |
|  | ____ | ____ | ____ | __ __ | ____ | ____ | ___ ___ | ____ | ____ | ____ |
|  | ____ | ____ | ____ | __ __ | ____ | ____ | ___ ___ | ____ | ____ | ____ |
|  | ____ | ____ | ____ | __ __ | ____ | ____ | ___ ___ | ____ | ____ | ____ |
|  | ____ | ____ | ____ | __ __ | ____ | ____ | ___ ___ | ____ | ____ | ____ |
|  | ____ | ____ | ____ | __ __ | ____ | ____ | ___ ___ | ____ | ____ | ____ |
|  | ____ | ____ | ____ | __ __ | ____ | ____ | ___ ___ | ____ | ____ | ____ |
|  | ____ | ____ | ____ | __ __ | ____ | ____ | ___ ___ | ____ | ____ | ____ |

| **SECTION 4: HOUSEHOLD SOCIODEMOGRAPHIC INFORMATION** | | | | | | |
| --- | --- | --- | --- | --- | --- | --- |
| *Identify head of household from Section 1: FB01* | |  | | | |  |
| **FD01** | What is your relationship to the head of household? | Relation (*select from list below*): [__\|__] | | | | If HH 🡪 skip to FD10 |
|  | *Codes for SI01: Relationship to head of household:   \| 01 Head  02 Spouse / Partner  03 Son / Daughter  04 Son-in-law / Daughter-In-Law \| 05 Grandchild  06 Parent  07 Parent-In-Law  08 Brother / Sister  09 Brother-in-law / Sister-In-Law \| 10 Uncle/Aunt  11 Niece / Nephew  12 Other relative  13 Adopted / Foster / StepchilD \| 14 Servant (Live-in)  96 Other (Not related)  99 DON’T KNOW \| \| --- \| --- \| --- \| --- \| | | | | | |
| **FD02** | What ethnicity is (*insert name of head of household*)? Or to what ethnic group does (*insert name of head of household*) belong? | | Darood (mareehan, majerteen, dhulbahantee)…………………….0  Digil and mirifle………………………………………………………...1  Dilir (gaadsan, fuquhumad bari) …………...……………….……….2  Other (specify)________________________________________  Don’t know…………………………………………………….……….88  Refused response………………………………………………….....99 | | |  |
| **FD03** | What is the highest level and grade or year of school (*insert name of head of your household*) completed? | | Preschool…………………….….……0  Primary………………………….…….1  Secondary……………………..……...2  Higher………………………..…….….3  Don’t know…………………………..88  Refused response………………….99 | | [__\|__] Grade  (fill 88 if don’t know, fill 99 if refused response) |  |
| **FD04** | What was the most recent occupation of (*insert name of head of household*) prior to displacement from Somalia? | | Domestic work ………………………….…….......………………………....0  Religious or community leader …………………………………..……..….1  Teacher or NGO employee …….…………………………..………...…….2  Medical or healthcare……………………………………..…………...…….3  Trader or Merchant…………………………………………………………..4  Agriculture/Farming…………………………………………………………..5  Fishing…………………………………………………………………………6  Masonry……………………………………………………………………….7  Other (specify)_____________________________________________  Don’t know…………………………………………………….…………...88  Refused response…………………………………………………..........99 | | |  |
| **FD05** | Is (*insert name of head of household*) working currently? | | No…………………………………………………..……………………….....0  Yes…………………………………………………..…………………………1  Don’t know…………………………………………………….…………...88  Refused response…………………………………………………..........99 | | | 🡪 skip to FD07 |
| **FD06** | What type of work does (*insert name of head of household*) currently do? | | Domestic work ………………………….…….......………………………....0  Religious or community leader …………………………………..……..….1  Teacher or NGO employee …….…………………………..………...…….2  Medical or healthcare……………………………………..…………...…….3  Trader or Merchant…………………………………………………………..4  Agriculture……………………………………………………………………..5  Other (specify)_____________________________________________  Don’t know…………………………………………………….…………...88  Refused response…………………………………………………..........99 | | |  |
| **FD07** | Where is (*insert name of head of household*) from originally in Somalia? | | Bay region..……………………………..………………………………….0  Bakool region.………………………………………………..…………….1  Geda regon.………………………………………………..……………….2  Shabelle region.………………………………………………..…………..3  Other (specify)____________________________________________  Don’t know…………………………………………………….…………...88  Refused response…………………………………………………..........99 | | |  |
| **FD08** | Would you describe (*insert name of head of household*)’s place of origin as urban or rural? | | Urban…………………………..………………………….…….………..….0  Rural………..………………………………………………………………..1  Don’t know…………………………………………………….…………...88  Refused response…………………………………………………..........99 | | |  |
| **FD09** | What is your ethnicity? Or to what ethnic group do you belong? | | Darood (mareehan, majerteen, dhulbahantee)…………………….0  Digil and mirifle………………………………………………………...1  Dilir (gaadsan, fuquhumad bari) …………...……………….……….2  Other (specify)________________________________________  Don’t know…………………………………………………….……….88  Refused response………………………………………………….....99 | | |  |
| **FD10** | What was the highest level of schooling you attended? | | Preschool…………………………………………………..…….….……0  Primary…………………………………………………..…….….……….1  Secondary…………………………………………………..…….….……2  Higher…………………………………………………..…….….………...3  Don’t know…………………………………………………..…….….……88  Refused response…………………………………………………..……99 | | |  |
| **FD11** | Have you ever worked outside the home, prior to coming to Ethiopia? | | No…………………………………………………..………..………………..0  Yes…………………………………………………..…………..…………….1  Don’t know…………………………………………………….…………...88  Refused response…………………………………………………..........99 | | | 🡪 skip to FD13 |
| **FD12** | What was kind of work did you do, prior to displacement from Somalia? | | Domestic work ………………………….…….......………………………....0  Religious or community leader …………………………………..……..….1  Teacher or NGO employee …….…………………………..………...…….2  Medical or healthcare……………………………………..…………...…….3  Trader or Merchant…………………………………………………………..4  Agriculture/Farming…………………………………………………………..5  Fishing…………………………………………………………………………6  Masonry……………………………………………………………………….7  Other (specify)_____________________________________________  Don’t know…………………………………………………….…………...88  Refused response…………………………………………………..........99 | | |  |
| **FD13** | Are you working currently? | | No…………………………………………………..………..………………..0  Yes…………………………………………………..…………..…………….1  Don’t know…………………………………………………….…………...88  Refused response…………………………………………………..........99 | | | 🡪 skip to FD15 |
| **FD14** | What was kind of work do you do now in Ethiopia? | | Domestic work ………………………….…….......………………………....0  Religious or community leader …………………………………..…..…….1  Teacher or NGO employee …….…………………………..………...….2  Medical or healthcare……………………………………..…………...….3  Trader or Merchant………………………………………………………..4  Agriculture………………………………………………………………….5  Other (specify)___________________________________________  Don’t know…………………………………………………….…………...88  Refused response…………………………………………………..........99 | | |  |
| **FD15** | Where are you from originally? | | Bay region..……………………………..………………………………….0  Bakool region.………………………………………………..…………….1  Geda regon.………………………………………………..……………….2  Shabelle region.………………………………………………..…………..3  Other (specify)____________________________________________  Don’t know…………………………………………………….…………...88  Refused response…………………………………………………..........99 | | |  |
| **FD16** | Would you describe your place of origin as a city or village? | | Urban…………………………..………………………….…….………..….0  Rural………..………………………………………………………………..1  Don’t know…………………………………………………….…………...88  Refused response…………………………………………………..........99 | | |  |
| **FD17** | The next question asks about difficulties doing certain activities because of a physical or mental disability. Does anyone in your household have moderate to severe difficulties? | | No…………………………………………………..………..………………..0  Yes…………………………………………………..…………..…………….1  Don’t know…………………………………………………..………...…….88  Refused response…………………………………………………………..99 | | | 🡪 skip to FD20 |
| **FD18** | Who experiences these difficulties? | | Me……………………………………………………………………………..0  My mother…………….………………………………………………………1  My father………….…………………………………………………………..2  A sibling………….……………………………………………………………3  My child………….……………………………………………………………4  Other (specify)___________________________________________  Don’t know………………………………………………………….....…….88  Refused response…………………………………………………………..99 | | |  |
| **FD19** | What kind of difficulty do they experience?  ***[Read responses to participant and select all that apply]*** | | Seeing, even if wearing glasses……………………………………………0  Hearing, even if using a hearing aid……………………………………….1  Walking or climbing steps……………………………………………………2  Remembering or concentrating……………………………………………..3  Caring for themselves, such as washing all over or dressing…………...4  Communicating, either expressing themselves or being understood…..5  None of the above……………………………………………………………6  Don’t know……………………………………………………..………...….88  Refused response…………………………………………………………..99 | | |  |
| **FD20** | How many times has this household moved within the past 12 months? | | [__\|__] | Times moved  (fill 88 if don’t know; fill 99 if refused response) | |  |

| **SECTION 5: MARRIAGE PERCEPTIONS** | | | | | | |
| --- | --- | --- | --- | --- | --- | --- |
| **FE01** | At what age did you, yourself, marry? | [__\|__] | | Years  (fill 88 if don’t know, fill 99 if refused response) | |  |
| **FE02** | At what age do you think young people in your community should marry? | **FEMALES** | | | **MALES** |  |
|  |  | [__\|__] Age (years)  (fill 88 if don’t know; fill 99 if refused response) | | | [__\|__] Age (years)  (fill 88 if don’t know; fill 99 if refused response) |  |
| **FE03** | Currently, in your community, what primarily influences decisions about the age people become married? | Religion…………………………………………….……………….……….0  Family honor……………………………………….………………………..1  Family tradition…………………………………….………………………..2  Money and/or resources……………………………….…………………..3  War and/or conflict…………………………………………….……………4  Displacement………………………………………………….…………….5  Other (specify) ___________________________________________  Don’t know………………………………………………..………...…..….88  Refused response…………………………………………………..……..99 | | | |  |
| **FE04** | Is this the same factor that influenced decisions about the age people became married prior to displacement? | No………………………………………………………………………..…..0  Yes…………………………………………………………………………..1  Don’t know………………………………………………..………...…..….88  Refused response…………………………………………………..……..99 | | | | 🡪 skip to FE06 |
| **FE05** | In your community, what primarily influenced decisions about the age people become married, prior to displacement? | Religion…………………………………………….……………….……….0  Family honor……………………………………….………………………..1  Family tradition…………………………………….………………………..2  Money and/or resources……………………………….…………………..3  War and/or conflict…………………………………………….……………4  Displacement………………………………………………….…………….5  Other (specify) ___________________________________________  Don’t know………………………………………………..………...…..….88  Refused response…………………………………………………..……..99 | | | |  |
| **FE06** | In your community today, who most significantly influences the age that a boy will marry?  **[Circle all that are mentioned]** | The boy……………………………………………………….…….…………0  The girl…………………………………………………………….………….1  The boy’s parents………………………………………………….………...2  The girl’s parents………………………………….………………………....3  The boy’s relatives……………………..………………………………..…..4  The girl’s relatives…………………………………………………..……….5  Other (specify)_____________________________________________  Don’t know………………………………………………..………...…..….88  Refused response…………………………………………………..……..99 | | | |  |
| **FE07** | In your community today, who most significantly influences the age at which a girl will marry?  **[Circle all that are mentioned]** | The boy……………………………………………………….…….……….…0  The girl…………………………………………………………….………..….1  The boy’s parents………………………………………………….…….…...2  The girl’s parents………………………………….…………………….…....3  The boy’s relatives……………………..………………………………..……4  The girl’s relatives…………………………………………………..….……..5  Other (specify)_____________________________________________  Don’t know………………………………………………..………...…..….88  Refused response…………………………………………………..……..99 | | | |  |
| **FE08** | In your community, is there typically an age difference between the bride and the groom? | No……………………………………………………………………………..0  Yes………………………………………………………………………..…..1  Don’t know………………………………………………..………...…..….88  Refused response…………………………………………………..……..99 | | | | 🡪 skip to FE10 |
| **FE09** | In your community, what is typically the age difference between the bride and the groom when they get married? | Man is older than bride by less than 5 years………………………………0  Man is older than the bride by 5-10 years………………………….……..1  Man is older than the bride by 10 years or more…………………………2  Don’t know………………………………………………..………...…..….88  Refused response…………………………………………………..……..99 | | | |  |
| **FE10** | What authority does this household trust to formalize marriage? | Government officials………..………………………….……..….…………..0  Religious leader …….………………………………….……..….……..…....1  Community leader……………………………………….……..….……..…..2  Parents/relatives of those engaged...…………………………..….……….3  Other (specify)_____________________________________________  Don’t know………………………………………………..………...……….88  Refused response…………………………………………………………..99 | | | |  |
| **FE11** | What is the legal, minimum age of marriage for girls? | [__\|__] | Years  (fill 77 if no law; 88 if don’t know; fill 99 if refused response) | | |  |
| **FE12** | What is the legal, minimum age for marriage for boys? | [__\|__] | Years  (fill 77 if no law; fill 88 if don’t know; fill 99 if refused response) | | |  |

| **SECTION 6: INTERVENTION EXPOSURE** | | | | |
| --- | --- | --- | --- | --- |
| **FF01** | Have you recieved any information or education about the effects of child marriage on girls? | No……………………………………………………………………………..0  Yes………………………………………………………………………..…..1  Don’t know………………………………………………..…………...…….88  Refused response…………………………………………………………..99 | |  |
| **FF02** | Have you ever participated in educational programs for parents or adolescents? | No…………………………………………………………………………..…0  Yes……………………………………………………………………..……..1  Don’t know………………………………………………..…………...…….88  Refused response…………………………………………………………..99 | | 🡪 skip to FF06 |
| **FF03** | What types of educational programs have you participated in?  ***[Circle all that are mentioned]*** | Life skills training………………………………………………….…………0  Mentorship program………………………………………………………....1  Community presentation………………………………………………....…2  Other (specify): ____________________________________________  Don’t know………………………………………………..…………...…….88  Refused response…………………………………………………………..99 | |  |
| **FF04** | What format did you receive this information?  ***[Circle all that are mentioned]*** | Pamphlet……………………………………………………………………...0  Community meeting……………...………………………………………….1  Radio………………………………………………………………………….2  Other (specify): ____________________________________________  Don’t know………………………………………………..…………...…….88  Refused response…………………………………………………………..99 | |  |
| **FF05** | Do you know which organization provided this information to you? | No……………………………………………………………………………..0  Yes………………………………………………………………………..…..1  Please specify the name of the organization: ___________________  Refused response…………………………………………………………..99 | |  |
| **FF06** | Have you ever received financial support—money or resources—to send your children to school? | No…………………………………………………………………………..…0  Yes……………………………………………………………………..……..1 | | 🡪 skip to FF08 |
| **FF07** | Do you know which organization provided this information to you? | No……………………………………………………………………………..0  Yes……………………………………………………………………..……..1  Please specify the name of the organization: __________________  Refused response…………………………………………………………..99 | |  |
| **FF08** | How many times in the past month have you attended the Women and Girls Center? | [__\|__] | Times  (fill 88 if don’t know; fill 99 if refused response) |  |
| **FF09** | Have you ever been told that marriage of children before the legal, minimum age is harmful? | No……………………………………………………………………………..0  Yes……………………………………………………………………..……..1 | |  |
| ***Read to respondent:*** *We are done with the survey. Thank you for your time today.* | | | |  |
